# Disseminated Gonococcal Infection With Dermatitis-Arthritis Syndrome

**DOI:** 10.7759/cureus.44991

**Published:** 2023-09-10

**Authors:** Sara Humayun, Atul Bali, Sreekant Avula, Roopa Naik

**Affiliations:** 1 Internal Medicine, Geisinger Health System, Wilkes-Barre, USA; 2 Internal Medicine / Nephrology, Geisinger Medical Center, Danville, USA; 3 Internal Medicine / Nephrology, Geisinger Health System, Wilkes-Barre, USA; 4 Medicine, Geisinger Commonwealth School of Medicine, Scranton, USA; 5 Endocrinology, Diabetes and Metabolism, University of Minnesota, Rochester, USA; 6 Internal Medicine / Hospital Medicine, Geisinger Health System, Wilkes-Barre, USA

**Keywords:** skin rash, arthritis, sexually transmitted infection, nucleic acid amplification test, naat

## Abstract

Gonorrhea is a sexually transmitted infection caused by gram-negative diplococci, *Neisseria gonorrhoeae*. Disseminated gonorrhea is diagnosed infrequently, partly due to low suspicion at the time of presentation, and at times, due to overlapping symptoms associated with non-infectious conditions like systemic lupus erythematosus (SLE). In this article, we present a 42-year-old sexually active female with knee pain and swelling, fever, and rash. Knee aspirate showed the presence of monosodium urate crystals, and the synovial culture grew gram-negative diplococci, requiring multiple joint washouts. The urine nucleic acid amplification test (NAAT) was indeterminate. She was treated with high-dose intravenous ceftriaxone for one week post-joint washout with rapid improvement in her condition and resolution of the rash.

## Introduction

Gonorrhea is caused by gram-negative diplococci, *Neisseria gonorrhoeae*. It is one of the most common reportable, sexually transmitted infections in the United States. Over 700,000 cases were reported to the CDC in 2021 although most strains remain susceptible to ceftriaxone [1]. While the typical gonorrheal infection leads to cervicitis in women and urethritis in men, untreated infections often result in pelvic inflammatory diseases, ectopic pregnancies, and infertility in both men and women [2]. The disseminated gonorrheal infection (DGI) is seen in less than 3% of all gonorrheal cases with complications ranging from polyarthritis to an overlap of polyarthritis with cutaneous manifestations [3], and in rare circumstances, endocarditis and meningitis have been reported [4,5].

This article was previously posted to the Authorea preprint server on June 7, 2023.

## Case presentation

**Figure 1:**
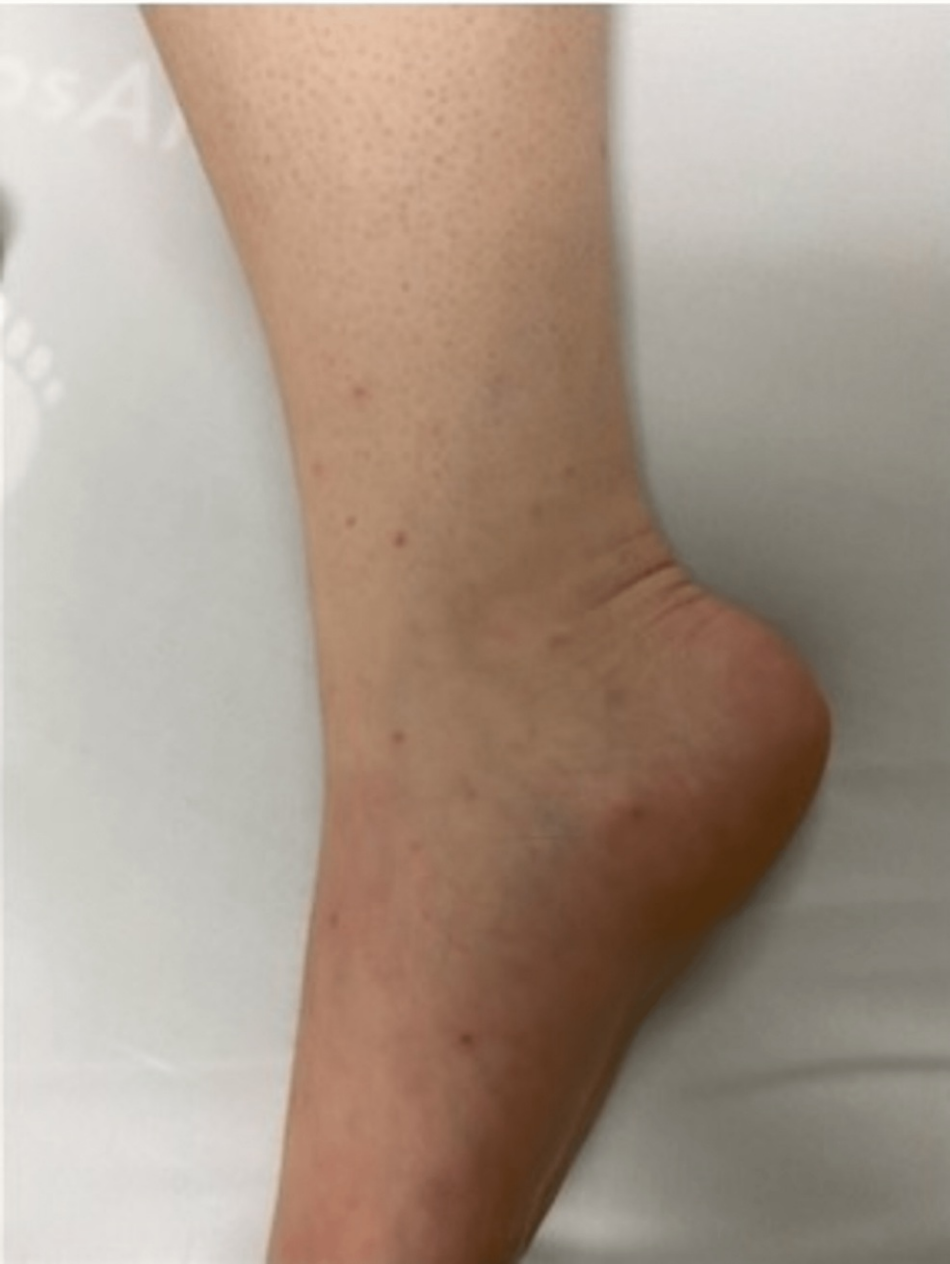
Lower extremity rash

**Figure 2:**
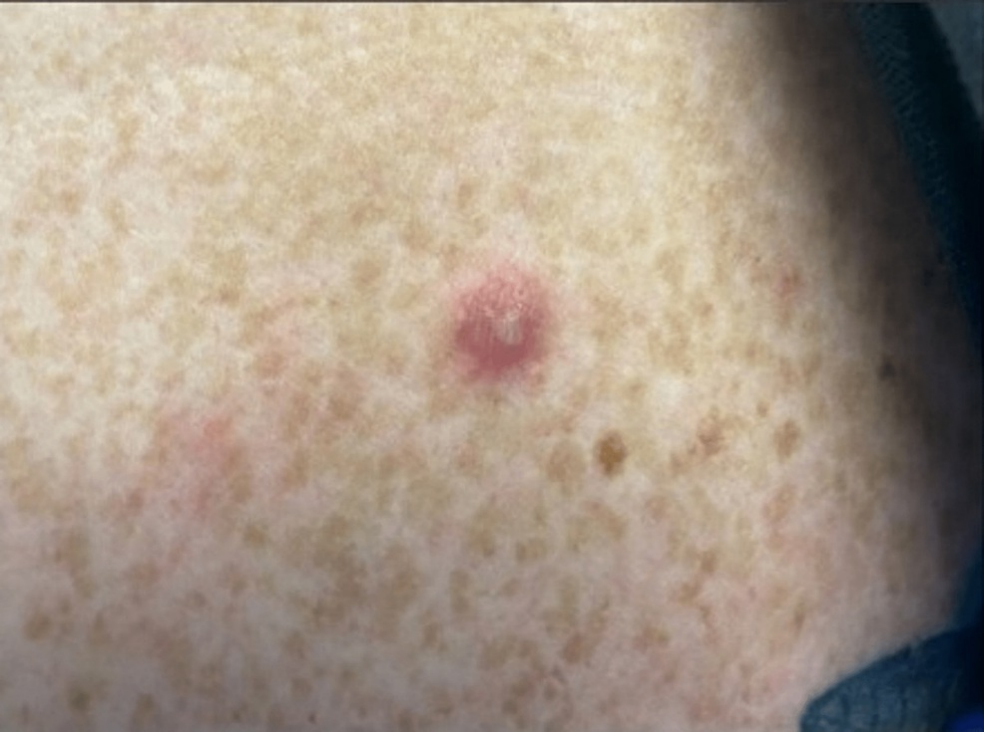
Vesiculopustular rash seen in disseminated gonococcal infection

A 42-year-old woman with a history of asthma and diet-controlled type 2 diabetes presented to the hospital with knee pain and swelling along with fever and rash for four days. Prior to admission, the patient developed a fever of 103.9 F followed by the development of a generalized non-pruritic rash predominantly in the lower extremities and subsequently developed polyarticular swelling and erythema in her right foot and bilateral knees. The patient reported unprotected sexual intercourse with a new partner a week prior to the presentation. Vitals on admission were blood pressure of 137/80 mmHg, pulse 88 beats per minute, temperature 97.8 F, respirations 18 breaths per minute, and oxygen saturation of 99% on room air. On examination, the skin showed the presence of diffuse vesiculopustular, non-tender, non-pruritic rashes (Figures 1, 2). The right foot was erythematous and associated with ankle edema, and the left knee exhibited mild swelling with a reduced range of motion due to tenderness. Labs on admission showed a white count of 21,000/microL. HIV antigen and antibody testing were negative. HbA1c was 8.2. Two sets of blood cultures were negative. The urine nucleic acid amplification test (NAAT) was indeterminate. She was started empirically on intravenous vancomycin and ceftriaxone. Right knee swelling confirmed joint effusion on imaging which was aspirated, revealing monosodium urate crystals in synovial fluid analysis and the culture growing gram-negative diplococci. As synovial fluid NAAT was not performed since the synovial cultures were positive. No cervical motion tenderness was noted. Disseminated gonococcal infection was suspected and the patient underwent recurrent (2 in 5 days) synovial joint washouts due to persistent symptoms of pain and reduced range of motion. The patient’s symptoms improved over the five-day hospital course and ultimately discharged home to continue ceftriaxone for a total of seven days post-final joint washout. The patient was encouraged to discuss her diagnosis with her recent partner to ensure appropriate treatment of the partner.

## Discussion

Gonorrhea is a sexually transmitted infection caused by *N. gonorrhoeae* with resultant genital infections, and rarely, pharyngeal and anorectal infections [6]. Bacteremia resulting in DGI is found to be more common during pregnancy, menstruation, and with the use of intra-uterine devices (IUDs) [7] and is seen in about 0.5-3% of the cases [8]. Other risk factors predisposing to dissemination include homosexual or bisexual men, infection with resistant strains, and complement deficiencies [9]. Eculizumab, a monoclonal antibody to complement protein 5, used for the treatment of complement-mediated conditions like hemolytic-uremic syndromes has been associated with an increased risk of gonococcal disease [10]. DGI is more common among females than males; this is likely due to a higher percentage of women remaining asymptomatic, hence delaying treatment, along with subsequent endometrial exposure of submucosal vessels to the bacteria, leading to the dissemination of infection [11].

Blood cultures are found to be positive in less than one-third of disseminated gonorrhea cases. Positive blood cultures are more often seen in patients that present with cutaneous involvement as well as gonococcal polyarthritis. Cutaneous manifestations in DGI along with polyarthritis can also be accompanied by tenosynovitis. Cutaneous findings are predominantly noted on the trunk and extremities. Our patient was found to have a generalized maculopapular, non-tender, and petechial rash. Joint involvement in DGI is manifested by polyarthralgia but never as suppurative arthritis [12].

Polyarthralgia is typically asymmetric and can affect all joints, large and small. Our patient was also a newly diagnosed diabetic which likely led to worsening of the underlying infection and presentation of cutaneous manifestations. A study of 55 patients with disseminated infections from *Neisseria gonorrhoeae* revealed that this complication occurs in young adults, with a predominance in women (80%) with arthritis being the most common manifestation [13].

*N. gonorrhoeae* is isolated in about 25% of the synovial fluid obtained by arthrocentesis. Synovial cultures are less sensitive in gonococcal arthritis compared to NAAT in the detection of *N. gonorrhoeae*. Whenever feasible, NAAT is recommended as the initial test for patients, including asymptomatic patients, with suspicion of gonorrhea. One case study concluded that when DGI is considered as part of the differential diagnosis, the gonococcal infection cannot be excluded solely on negative urine NAAT. A thorough history, which includes sexual history, testing of appropriate sites (rectal, oropharyngeal) along with NAAT of the joint aspirate, should be considered when suspicion is high [5]. Cautious interpretation of a negative NAAT must be done since some *N. gonorrhoeae* subtypes can have variations in the targeted sequences tested by NAAT resulting in a false-negative test [14].

DGI has been reported in patients with systemic lupus erythematosus (SLE); likely mechanisms include complement depletion, the use of immunosuppressive medications, and an association between SLE and inherited complement disorders [15]. Given the overlap of symptoms between DGI and SLE, a high degree of suspicion is warranted to diagnose DGI in patients with SLE [16].

## Conclusions

Early detection and appropriate treatment of disseminated gonorrhea with cutaneous and joint involvement is the key to the prevention of lifelong disability. Given its rare presentation, a low threshold must be held for prompt testing of gonorrhea and initiation of treatment, especially in patients with SLE and immunosuppressed states. A detailed sexual history is indicated in all the patients when DGI is considered a possible diagnosis to avoid delays in the diagnosis and treatment. If DGI is suspected and considered a likely differential, a negative urine NAAT should not dissuade the clinicians from pursuing further diagnostic tests.
